# Vector Competence of *Aedes aegypti* and *Aedes polynesiensis* Populations from French Polynesia for Chikungunya Virus

**DOI:** 10.1371/journal.pntd.0004694

**Published:** 2016-05-04

**Authors:** Vaea Richard, Tuterarii Paoaafaite, Van-Mai Cao-Lormeau

**Affiliations:** Institut Louis Malardé, Papeete, Tahiti, French Polynesia; United States Army Medical Research Institute of Infectious Diseases, UNITED STATES

## Abstract

**Background:**

From October 2014 to March 2015, French Polynesia experienced for the first time a chikungunya outbreak. Two *Aedes* mosquitoes may have contributed to chikungunya virus (CHIKV) transmission in French Polynesia: the worldwide distributed *Ae*. *aegypti* and the Polynesian islands*-*endemic *Ae*. *polynesiensis* mosquito.

**Methods:**

To investigate the vector competence of French Polynesian populations of *Ae*. *aegypti* and *Ae*. *polynesiensis* for CHIKV, mosquitoes were exposed *per os* at viral titers of 7 logs tissue culture infectious dose 50%. At 2, 6, 9, 14 and 21 days post-infection (dpi), saliva was collected from each mosquito and inoculated onto C6/36 mosquito cells to check for the presence of CHIKV infectious particles. Legs and body (thorax and abdomen) of each mosquito were also collected at the different dpi and submitted separately to viral RNA extraction and CHIKV real-time RT-PCR.

**Results:**

CHIKV infection rate, dissemination and transmission efficiencies ranged from 7–90%, 18–78% and 5–53% respectively for *Ae*. *aegypti* and from 39–41%, 3–17% and 0–14% respectively for *Ae*. *polynesiensis*, depending on the dpi. Infectious saliva was found as early as 2 dpi for *Ae*. *aegypti* and from 6 dpi for *Ae*. *polynesiensis*. Our laboratory results confirm that the French Polynesian population of *Ae*. *aegypti* is highly competent for CHIKV and they provide clear evidence for *Ae*. *polynesiensis* to act as an efficient CHIKV vector.

**Conclusion:**

As supported by our findings, the presence of two CHIKV competent vectors in French Polynesia certainly contributed to enabling this virus to quickly disseminate from the urban/peri-urban areas colonized by *Ae*. *aegypti* to the most remote atolls where *Ae*. *polynesiensis* is predominating. *Ae*. *polynesiensis* was probably involved in the recent chikungunya outbreaks in Samoa and the Cook Islands. Moreover, this vector may contribute to the risk for CHIKV to emerge in other Polynesian islands like Fiji, and more particularly Wallis where there is no *Ae*. *aegypti*.

## Author Summary

Chikungunya virus has caused a series of outbreaks in the Pacific from 2011, including French Polynesia. *Aedes* (*Ae*.) *aegypti* mosquito, which has colonized almost all Pacific Island Countries, is reasonably expected to have been involved in the chikungunya outbreaks. In addition, endemic *Aedes* mosquito species may have sustained chikungunya virus transmission in the less urbanized and most remote islands. In the present study, we demonstrated the ability of French Polynesian populations of *Ae*. *aegypti* and *Ae*. *polynesiensis* to replicate, disseminate and transmit chikungunya virus under experimental conditions. Our results provide for the first time clear evidence for *Ae*. *polynesiensis* to act as an efficient chikungunya vector. These findings corroborate previous observation that endemic *Aedes* species, like *Ae*. *hensilli* in Yap Island, may play a critical role in sustaining chikungunya virus transmission, in place or together with the widely distributed *Ae*. *aegypti* and *Ae*. *albopictus*. In a context where innovative vector control strategies are mostly focused on targeting the mosquito species considered as the main arbovirus vectors, the potential for endemic *Aedes* species to take the lead in transmitting such arboviruses should not be neglected.

## Introduction

Chikungunya virus (CHIKV; *Togaviridae*: *Alphavirus*) infection usually produces fever, joint pain, maculopapular rash and chronic polyarthralgia [1].Since its emergence in the Indian Ocean islands in 2005, CHIKV has caused a series of outbreaks in the Indian subcontinent, South-East Asia, China and Central Africa, and, following an increasing trend, CHIKV also expanded to countries in Europe, the Pacific, the Caribbean and the Americas [2–4].

CHIKV is a single-stranded positive sense RNA virus that genetically has diverged in four lineages: the three original West African, East Central South African (ECSA) and Asian lineages; and the new ECSA-derived Indian Ocean lineage (IOL) [2].

French Polynesia is a French overseas Territory of about 270 000 inhabitants, located in the East part of the South Pacific Ocean. Until October 2013 and the first appearance of Zika virus (*Flaviviridae*: *Flavivirus*), dengue virus (*Flaviviridae*: *Flavivirus*) used to be the only arbovirus formally proven as circulating in French Polynesia [5]. From October 2014 to March 2015, French Polynesia experienced its first CHIKV outbreak. Within a few weeks CHIKV transmission expanded to all the districts on the main island Tahiti and then rapidly to several islands in all five archipelagos of French Polynesia (Society, Marquesas, Tuamotu, Gambier and Austral Islands). As of March 2015, 69,000 suspected CHIKV cases had been recorded by the Direction of Health [4,6]. Phylogenetic analysis confirmed that the virus was introduced from the Caribbean and that it belonged to the Asian lineage [7].

CHIKV is transmitted by daytime-biting *Aedes* mosquitoes, mostly the wide distributed *Ae*. *aegypti*, but also *Ae*. *albopictus* that is able to survive at temperate climates [8–14]. Several other *Aedes* mosquito species have also been reported as potential vectors for sylvatic transmission of CHIKV in Africa and Asia [15].

In the Pacific region, *Ae*. *aegypti* started colonizing the islands in the late 19^th^ and early 20^th^ centuries. In the late 1930s infestations were reported in the North part of the Pacific (Guam, Palau, Federated States of Micronesia, Marshall Islands…) and also in Vanuatu (New Hebrides) and in the Solomon Islands [16,17]. *Ae*. *aegypti* is now present in almost all Pacific islands and because its ability to transmit CHIKV had been demonstrated, the Pacific island countries were considered at high risk for CHIKV to emerge [14]. In 2011, CHIKV was reported for the first time in New Caledonia and local populations of *Ae*. *aegypti* were demonstrated as able to transmit CHIKV [11].

In Pacific islands or remote areas where *Ae*. *aegypti* is not or poorly present, CHIKV may have been transmitted by endemic *Aedes* species, like *Ae*. *hensilli* in Yap State in 2013 [18]. In French Polynesia, possible contribution of the endemic *Ae*. *polynesiensis* species in CHIKV transmission was suspected. *Ae*. *polynesiensis* may have settled in the Polynesian islands together with human population migrations from the far west to the east part of the Pacific approximately 1,500–3,000 years ago [19]. Because the *Ae*. *polynesiensis* gravid adult female preferentially looks for natural breeding sites such as coconut shells, tree-holes or crab-holes and because its larvae can develop in brackish water, this mosquito is widely distributed in the Polynesian islands [19–22]. In the 1980s after the occurrence of several outbreaks caused by Ross River virus (RRV; *Togaviridae*: *Alphavirus)* in Pacific islands, the ability for *Ae*. *polynesiensis* to transmit RRV was investigated and demonstrated [3,23]. These observations suggested *Ae*. *polynesiensis* may also be able to transmit CHIKV. In 1967, Gilotra and Shah mentioned for the first time the ability of a Samoan population of *Ae*. *polynesiensis* to experimentally transmit CHIKV [9].

In the present study, we investigated the vector competence of French Polynesian populations of *Ae*. *aegypti* and *Ae*. *polynesiensis* for CHIKV.

## Materials and Methods

### Mosquito Rearing

*Ae*. *aegypti* and *Ae*. *polynesiensis* mosquito colonies were established in 2014, using mosquito collected on Tahiti Island in the districts of Toahotu and Atimaono, respectively. For the purpose of the study, F14-generation eggs of each mosquito colony were hatched under negative pressure in tap water for 1 hour. Larvae were reared in plastic trays containing tap water supplemented with bovine liver powder (MP Biomedicals, USA) inside a climate chamber (Sanyo MLR-351H, Japan) set at 27°C, 80% relative humidity and 12:12h light-dark cycle. Pupae were selected with a ratio of 1 male: 4 females. Adults were then maintained in climatic conditions as indicated above and were given continuous access to 10% sucrose solution.

### Virus

CHIKV strain PF14/300914-109 was isolated from the serum of patient infected in September 2014 in Tahiti, French Polynesia. Amplification of CHIKV was performed by inoculation of *Ae*. *albopictus* C6/36 cells [24] routinely maintained at 30°C in RPMI-1640 medium supplemented with non essential amino acids, gentamicin, fungizone (Amphotericin B) and 10% heat-inactivated foetal bovine serum (FBS, Life technologies, USA). Serum was inoculated at 1:40 in cell-culture medium adjusted at 1% of FBS for 30 minutes at 30°C. Inoculum was then removed and replaced by fresh 1% FBS cell-culture medium. Infected cells were incubated at 30°C for 4 days. Infected cell-culture supernatant was then harvested and underwent two successive additional passages on C6/36 cells. Each successive passage was performed as follows: infected cell-culture supernatant from the previous passage was inoculated at 3:1 in 1% FBS-cell-culture medium for 1 hour at 37°C with gentle agitation. The inoculum was then replaced by fresh 1% FBS-cell-culture medium and infected cells were incubated at 30°C for 4 days. After the third passage, the infected-cell supernatant was harvested and concentrated by using Centricon Plus-70 centrifugal filter devices (Millipore, Germany) as previously described [25]. FBS was added to the CHIKV concentrate at 1:5 before storage at -80°C.

Virus titration was performed by inoculating C6/36 cells with serial 10-fold dilutions of virus concentrate on a 96-wells plate. Six days later, C6/36 cells were fixed directly on the plate with 70% ice-cold acetone for 10 minutes. Each well was then incubated 30 minutes at 37°C with Group-A mouse ascitic fluid (National Institute of Allergy and Infectious Diseases, USA) diluted 1:100 in PBS followed by 30 minutes incubation at 37°C with fluorescein isothiocyanate-conjugated goat anti-mouse IgG (Bio-Rad Laboratories, France) diluted 1:100. Wells containing infected cells were counted and viral titers in 50% tissue culture infectious dose (TCID50/mL) were calculated using the method of Reed and Muench [26].

### Mosquito Infection

The day of infection, 24 hours-starved and water-deprived 5-days-old mosquitoes were transferred into four to eight nylon mesh-covered containers of about 70 mosquitoes for each population.Two hundred *Ae*. *aegypti* mosquitoes were offered the CHIKV infectious blood meal. For *Ae*. *polynesiensis*, as the survival rate in laboratory conditions seemed to be lower than for *Ae*. *aegypti*, >400 *Ae*. *polynesiensis* females were offered the meal to ensure getting enough mosquitoes surviving at least 9 days later.

The infectious meal was prepared with fresh washed bovine red cells, viral concentrate (1:22) and adenosine triphosphate at 5 mM as phagostimulant. As used in previous studies CHIKV titer in the blood meal was adjusted to 7 log_10_ TCID_50_/mL to be close to the viremia levels observed in patients [10,11,27].

Blood meal maintained at 37°C was offered through a Parafilm-M membrane to *Ae*. *aegypti* mosquitoes and through a porcine membrane to *Ae*. *polynesiensis* mosquitoes. After 1 hour of free access to the blood meal each fully-engorged female was transferred into a 67 x 26 mm individual plastic container to avoid horizontal transmission during sugar-feeding [28,29]. Mosquitoes were given access to 10% sucrose solution and maintained for up to 21 days in the climate chamber set at 27°C, 80% relative humidity and 12:12h light-dark cycle.

### Saliva Collection

At days 2, 6, 9, 14 and 21 after the infectious blood meal, a subset of 18 hours sucrose-starved and water-deprived mosquitoes were cold-anesthetized. Legs and wings from each mosquito were carefully removed and the proboscis was inserted into an individual filter tips ART (Molecular BioProducts, USA) containing 20 μL of FBS. Mosquitoes were allowed to expectorate saliva for 30 min. Then the FBS was expelled into a microtube containing 80 μL of 1% FBS cell-culture medium and stored at -80°C until tested. Each saliva sample was inoculated to C6/36 cells in a single well of a 96-well plate and 6 days later, infectious wells were determined by indirect immunofluorescent assay as described above.

### Mosquito Dissection

At days 2, 6, 9, 14 and 21 after the infectious blood meal, legs and body (thorax and abdomen) of each mosquito were collected in separate microtubes and stored at -80°C until tested.

### RNA Extraction and Reverse Transcription Polymerase Chain Reaction (RT-PCR)

Individual mosquito legs and bodies were separately homogenized with metal beads at 20 Hz for 4 min (Mixer Mill Retsch MM301, Germany) either in cell-culture medium supplemented at 20% FBS for bodies or directly in NucliSENS lysis buffer (bioMérieux, France) for legs. Homogenate supernatants were recovered after centrifugation at 20,000 x g during 5 minutes. Viral RNA was extracted using NucliSENS miniMAG system (bioMérieux, France) according to manufacturer’s instructions. Real time RT-PCR was processed on a CFX96 Touch Real-Time PCR Detection System instrument using iScript One-Step RT-PCR Kit for Probes (Bio-Rad Laboratories, France). The primers and the probe used were previously described [30].

### Data and Statistical Analysis

Vector competence is defined as the ability of a mosquito to be infected, to disseminate and finally to be able to transmit a given virus [31]. Information on the ability of the two populations of mosquitoes to get infected was provided by the detection of CHIKV by RT-PCR performed on bodies. The mosquito infection rate was defined as the number of mosquitoes with positive body divided by the number of females tested at each time point.The aptitude of the two mosquito species to disseminate the virus was based on the detection of CHIKV by RT-PCR performed on legs. The viral dissemination efficiency was defined as the number of mosquitoes with positive legs divided by the number of females tested at each time point. Evidence for the potential for each of the species to be able to transmit the virus was given by the detection of replicative CHIKV particles in mosquito saliva. The viral transmission rate was defined as the number of mosquitoes with infectious saliva divided by the number of females tested at each time point.

Chi-square test with or without Yates’ correction or Fisher’s exact test were used to assess the differences between the two *Aedes* species at each time point and between two time points for each species (Graph Pad Prism software, USA).

## Results

### Mosquito Engorgement and Mortality Rate in CHIKV *Per Os* Infection Experiments

In our infection experiments, ~90% of *Ae*. *aegypti* and 70% of *Ae*. *polynesiensis* females were fully-engorged with the blood meal (Table 1). The mortality rate of initially fully-engorged females was much higher for *Ae*. *polynesiensis* compared to *Ae*. *aegypti* (Table 1). At 9 dpi, only 29 *Ae*. *polynesiensis* females had survived and all were sacrificed for this collecting day.

**Table 1:** Number of engorged mosquito females obtained the day of infection and mortality rate during the following days.

	Number of engorged females / N (% of females engorged)	Number of dead females / n (% of mortality)				
		0–2 dpi	2–6 dpi	6–9 dpi	9–14 dpi	14–21 dpi
***Ae*. *aegypti***	243/272 (89%)	7/243 (3%)	8/198 (4%)	11/150 (7%)	3/101 (3%)	10/59 (17%)
***Ae*. *polynesiensis***	295/422 (70%)	30/295 (10%)	98/228 (43%)	63/92 (68%)	-	-

N, number of females allowed feeding on CHIKV infectious blood-meal; n, number of females remaining from the previous period minus the number of females sacrificed for testing on the previous sampling day; dpi, day post-infection. A dash (-) indicates that there were no more female at these collecting days.

### CHIKV infection rate, dissemination and transmission efficiencies

The infection rate was calculated at different time post-infection except at 2 dpi to prevent any false positive RT-PCR due to remaining infectious blood meal in the mosquito midgut. The mosquito infection rate was ~80% as soon as 6 dpi for *Ae*. *aegypti* and ~40% for *Ae*. *polynesiensis* (Table 2). The infection rate was significantly higher for *Ae*. *aegypti* compared to *Ae*. *polynesiensis* (p<0.001 at 6 dpi and p<0.0001 at 9 dpi).

**Table 2:** CHIKV infection rate, dissemination and transmission efficiencies at 2, 6, 9, 14 and 21 days post-infection.

		Day 2	Day 6	Day 9	Day 14	Day 21
**Number of infected bodies / number of mosquitoes tested** (% of infection)	***Ae*. *aegypti***	nd/38	31/40 (78%)	33/38 (87%)	35/39 (90%)	32/40 (80%)
	***Ae*. *polynesiensis***	nd/37	15/38 (39%)***	12/29 (41%) ****	-	-
**Number of infected legs / number of mosquitoes tested** (% of dissemination)	***Ae*. *aegypti***	7/38 (18%)	14/40 (35%)	26/38 (68%)	28/39 (72%)	31/40 (78%)
	***Ae*. *polynesiensis***	1/37 (3%)	5/38 (13%)*	5/29 (17%)****	-	-
**Number of infectious saliva / number of mosquitoes tested** (% of transmission)	***Ae*. *aegypti***	2/38 (5%)	7/40 (18%)	13/38 (34%)	19/39 (49%)	21/40 (53%)
	***Ae*. *polynesiensis***	0/37 (0%)	1/38 (3%)	4/29 (14%)	-	-

Infection and dissemination were determined by real-time RT-PCR. Transmission was evaluated by inoculation of saliva on C6/36 cells to detect infectious particles of CHIKV. For collecting day 2, the number of infected bodies was not determined (nd) due to remaining blood-meal in midgut. Statistically significant differences between the two species are shown by asterisks (* = p<0.05; *** = p<0.001; **** = p<0.0001). A dash (-) indicates that females were not obtained for these collecting days.

**Fig 1:**
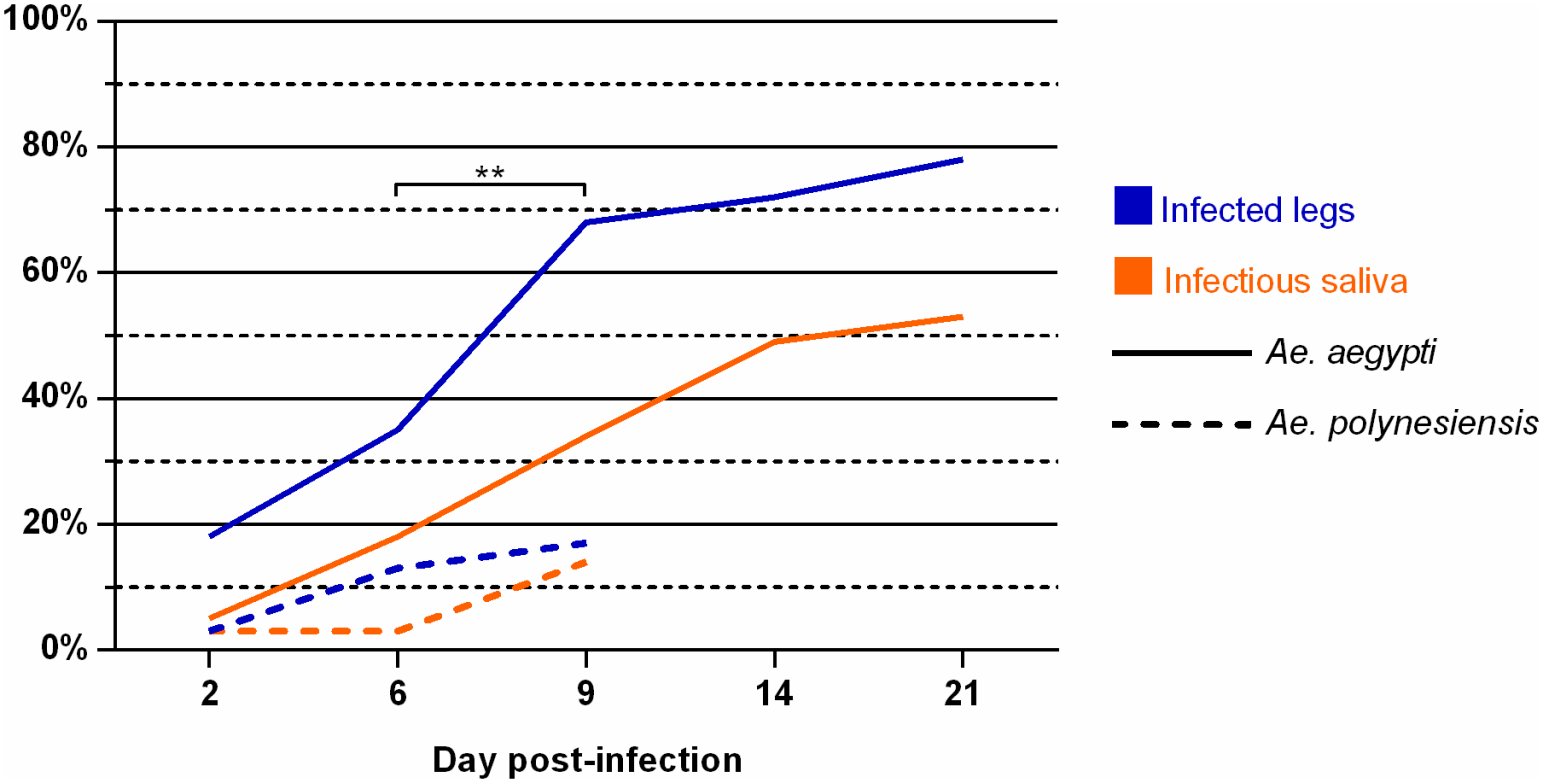
Progression trends of CHIKV dissemination and transmission efficiencies in *Ae*. *aegypti* and *Ae*. *polynesiensis*. Statistically significant differences between two successive days post-infection are shown by asterisks (** = p<0.01). CHIKV dissemination efficiency showed a dramatic increase in *Ae*. *aegypti* from 6 to 9 dpi.

At 2 dpi, CHIKV was detected in legs from seven *Ae*. *aegypti* and one *Ae*. *polynesiensis* mosquito, on 38 and 37 females tested respectively (Table 2). CHIKV dissemination efficiencies increased over days and especially between 6 and 9 dpi in *Ae*. *aegypti* (p<0.01; Fig 1). At 6 and 9 dpi, the dissemination rates in legs were significantly higher for *Ae*. *aegypti* compared to *Ae*. *polynesiensis* (p<0.05 and p<0.0001 respectively; Table 2). We observed at 6 dpi that RT-PCR cycle threshold values (data in S1 Table) in bodies of *Ae*. *aegypti* mosquitoes with negative dissemination were significantly higher than those in mosquitoes with positive dissemination (p<0.0001, Mann Whitney test).

Infectious saliva was detected as early as 2 dpi in two *Ae*. *aegypti* females and at 6 dpi in one *Ae*. *polynesiensis* female (Table 2). At 9 dpi, transmission rate was 34% for *Ae*. *aegypti* and 14% for *Ae*. *polynesiensis*. CHIKV transmission efficiency in *Ae*. *aegypti* increased regularly up to 14 dpi and then plateaued to reach 53% at 21 dpi (Fig 1).

## Discussion

In the present study we provided evidence that *Ae*. *aegypti* and *Ae*. *polynesiensis* from French Polynesia were competent laboratory vectors of CHIKV. We observed that the French Polynesian population of *Ae*. *aegypti* displayed CHIKV infection rates similar to those previously reported for *Ae*. *aegypti* populations collected in other countries [15]. As previously reported for an *Ae*. *aegypti* population from Mayotte, Comoros archipelago, infectious saliva was detected in the French Polynesian *Ae*. *aegypti* as early as 2 dpi [10]. Such a short extrinsic incubation period allows the vector to quickly infect susceptible people in the household of an infected patient. Together with the observation that dissemination and transmission efficiencies increased up to 21 dpi, our results support that *Ae*. *aegypti* may have been an efficient vector of CHIKV during the outbreak in French Polynesia. For *Ae*. *polynesiensis*, although the latter time points (>9 dpi) were not available, we found that CHIKV infection and dissemination efficiencies were lower compared to *Ae*. *aegypti*. Nevertheless, as vectorial capacity relies on multiple factors (mosquito densities, feeding behavior…) *Ae*. *polynesiensis* might have been able to sustain CHIKV transmission in areas where *Ae*. *aegypti* is poorly represented. Indeed, CHIKV spread very quickly in remote French Polynesian atolls, but also recently caused outbreaks in Pacific islands where *Ae*. *polynesiensis* is dominating, like in the Cook Islands and Samoa [20,22].

## Supporting Information

S1 Table Cycle threshold (Ct) values in bodies of *Ae*. *aegypti* mosquitoes at 6 dpi. (DOCX)
