# Supplementary material for: Vector Competence of Aedes aegypti and Aedes polynesiensis Populations from French Polynesia for Chikungunya Virus
Source: PLoS Negl Trop Dis. 2016 May 4;10(5):e0004694. doi: 10.1371/journal.pntd.0004694 (PMC4856362; doi:10.1371/journal.pntd.0004694)
Supplement: S1 Table — (DOCX) [file pntd.0004694.s001.docx]

**S1 Table. Cycle threshold (Ct) values in bodies of *Ae. aegypti* mosquitoes at 6 dpi.**

| **Ct values in bodies** (threshold 100) | |
| --- | --- |
| **Negative dissemination in legs** | **Positive dissemination in legs** |
| 25,43 | 19,23 |
| 23,25 | 20,46 |
| 23,74 | 19,88 |
| 25,23 | 19,25 |
| 23,78 | 19,33 |
| 24,48 | 21,76 |
| 25,81 | 25,28 |
| 25,87 | 20,51 |
| 25,46 | 20,94 |
| 24,93 | 20,08 |
| 23,71 | 21,84 |
| 24,39 | 20,31 |
| 23,96 | 24,84 |
| 26,66 | 22,35 |
| 27,81 |  |
| 25,57 |  |
| 25,69 |  |
